# Fungal L-Asparaginase from environmental isolates with antimicrobial and antibiofilm activities

**DOI:** 10.1186/s13568-026-02025-5

**Published:** 2026-03-19

**Authors:** Basma A. Khalifa, Zeinab S. Hashem, Mohamed Hisham, Emad H. Khedr, Shimaa M. Abdelhameed

**Affiliations:** 1 Botany and Microbiology Department, Faculty of Science, Minia University, Minia, Egypt; 2 Microbiology and Immunology Department, Faculty of Pharmacy, Minia University, Minia, Egypt; 3 Pharmaceutical Chemistry Department, Faculty of Pharmacy, Deraya University, New Minia, Egypt; 4 Pomology Department, Faculty of Agriculture, Cairo University, Giza, Egypt

**Keywords:** Sewage, Soil, Enzyme optimization, Cytotoxicity, Molecular docking

## Abstract

Fourteen fungal species were isolated from sewage water and soil, of which ten strains showed positive extracellular L-asparaginase production by plate assay. Among them, *Aspergillus nidulans* AUMC17371, *A. flavus* AUMC17373, *A. flavus* AUMC17374, and *A. terreus* AUMC17372 exhibited the highest enzyme activities (1.35, 1.25, 0.97, and 0.79 U/mL, respectively). Optimization of culture conditions revealed that maximum production was achieved using fructose as carbon source, asparagine as nitrogen source, pH 6, and 30 °C. SDS-PAGE analysis confirmed enzyme production, with protein bands ranging from 35 to 45 kDa. Antimicrobial screening revealed strong activity against Gram-positive and Gram-negative bacteria; however, only limited antifungal effectiveness against *Candida albicans* was exhibited. MIC results showed that L-asparaginase from *A. nidulans* AUMC17371 showed the strongest antibacterial effect against *Staphylococcus aureus*, followed by *A. flavus* AUMC17373 against *S. aureus* and *Pseudomonas aeruginosa*. Antibiofilm assays indicated inhibition ranging from 22 to 69%, with *A. flavus* AUMC17373 showing the most potent effect. Cytotoxicity testing revealed that *A. flavus* AUMC17374 enzyme exhibited anticancer activity against MCF-7 and HepG2 cell lines with IC_50_ values of 184 and 450 µg/mL, respectively. Computational analysis supported these findings, with molecular docking demonstrating stable binding of L-asparagine to *A. nidulans* AUMC17371 L-asparaginase (− 4.67 kcal/mol), and molecular dynamics indicating a structurally stable enzyme–substrate complex. Collectively, these results suggest that fungal L-asparaginase may serve as a promising candidate with antimicrobial, antibiofilm, and cytotoxic activities.

## Introduction

Cancer is one of the most devastating diseases worldwide. Lung cancer is the most frequently diagnosed form, while acute lymphoblastic leukemia (ALL) ranks as the most prevalent pediatric cancer (Bray et al. 2018). As an amidohydrolase enzyme, L-asparaginase is of great interest because of its antineoplastic capacity and its established role as a chemotherapeutic drug against ALL, and is also applied in treating chronic lymphocytic leukemia, acute myelomonocytic, and lymphosarcoma (Egler et al. 2016). L-asparaginase catalyzes the breakdown of L-asparagine into L-aspartate and ammonia, a reaction that is largely irreversible under physiological conditions. Its mechanism of action relies on depriving leukemic cells of L-asparagine. While normal cells can synthesize L-asparagine via asparagine synthetase, leukemic cells rely on extracellular sources. By reducing the available asparagine, L-asparaginase inhibits protein and RNA synthesis, causing cell cycle arrest and apoptosis (Shrivastava et al. 2016). Additionally, L-asparaginase possesses significant applications in the food industry, where it helps reduce acrylamide formation during thermal food processing (Dange et al. 2018).

Beyond its direct anticancer use, L-asparaginase research intersects with another major global challenge, namely antibiotic resistance. Excessive and inappropriate antibiotic use has led to resistant microbial strains, with World Health Organization projecting up to ten million deaths annually by 2050 if the current situation continues (Davies and Davies 2010). Moreover, the development of bacterial biofilms exacerbates this challenge by rendering bacteria highly tolerant to antibiotics (Roychoudhury et al. 2024). These bacteria contribute to persistent infections associated with medical devices and chronic diseases. Recent studies indicate that fungal extracts and natural products offer promising antimicrobial and antibiofilm activities (Choi et al. 2021; Roychoudhury et al. 2024).

The possible mechanism underlying the anti-infective action of L-asparaginase may involve its interaction with penicillin-binding proteins, which play a crucial role in bacterial cell wall biosynthesis (Vimal and Kumar 2021). L-asparaginase is speculated to exert bactericidal effects through a mechanism similar to that of lysozyme, which compromises bacterial cell wall integrity. Lysozyme achieves this by hydrolyzing the glycosidic bond between N-acetylmuramic acid and N-acetylglucosamine, ultimately leading to cell lysis (Vermassen et al. 2019). Likewise, L-asparaginase may disrupt the bacterial cell wall, contributing to its antimicrobial activity. Furthermore, the enzyme has also demonstrated antiviral potential, as evidenced by its inhibitory effect against Coxsackie B3 Virus, highlighting its promise as a broad-spectrum antimicrobial agent (Abd El-Baky and El-Baroty 2020). In addition to its antimicrobial effects, L-asparaginase has shown promising antibiofilm activity. Most conventional antibiofilm agents target the bacterial physical characteristics, whereas this enzyme modulates bacterial gene expression involved in biofilm development, thereby reducing biofilm stability with high efficiency (Darvishi et al. 2022).

L-asparaginase can be derived from diverse sources, such as fungi, bacteria, actinomycetes, and plants. Many fungi, such as *A. niger*, *A. terreus*, and *Penicillium cyclopium* have been reported to produce this enzyme (Souza et al. 2017). Microbial sources have attracted the most research attention due to their advantages, such as facilitated extraction and purification processes, easier optimization of culture conditions, growth on simple substrates, and higher enzyme stability compared to plant or animal derived enzymes (Parashiva et al. 2023). Environmental sources like soil, sewage, and medicinal plants are rich in microbial diversity that can produce L-asparaginase, which has important therapeutic and biotechnological uses (Borah 2012; Priya and Subhashini 2022). Isolation of fungi that produce L-asparaginase with reduced L-glutaminase activity from soil samples, emphasizing that microbial isolates from soil can yield enzymes with better therapeutic characteristics than those from conventional bacterial sources (Sisay et al. 2024). Accordingly, this study seeks to obtain L-asparaginase from various fungal strains isolated from sewage and soil samples, determine its activity and investigate its medical relevance. Few studies have concurrently combined environmental isolation, enzyme production, antimicrobial and antibiofilm evaluation, and computational validation of enzyme–substrate interactions, despite the fact that many have examined L-asparaginase production from fungal isolates and their biological activities. To our knowledge, this integrated approach is presented here for the first time, providing a comprehensive evaluation of fungal L-asparaginase potential for biomedical applications.

## Materials and methods

### Isolation and characterization of fungi from soil and sewage wastewater samples

Sterile containers were used to collect five wastewater samples (250 mL each) from the Wastewater Treatment Plant in Minia Governorate, Egypt. The plate-pouring method was applied, wherein one millilitre of each water sample was transferred into a sterile Petri dish. Sterilized Potato Dextrose Agar (PDA) was subsequently added, along with chloramphenicol at a concentration of 25 µg/mL. The plates were gently rotated to ensure adequate mixing. Two soil samples were homogenized in sterile saline solution (10 g in 90 mL) and underwent serial dilution to 10^-4^, 10^-5^, 10^-6^, and 10^-7^, according to the procedures outlined by (Kawaguchi et al. 2013), after which one millilitre of each dilution was spread on PDA plates supplemented with ampicillin (50 µg/mL) to suppress bacterial growth. All plates were then incubated at 30 °C for 7 days.

Fungal colonies were isolated, purified, and taxonomically identified to the genus level utilizing the identification keys provided by (Watanabe 2002), based on morphological traits, including colony colour, texture, and microscopic examination of spore formation. Microscopic assessment of hyphae and spores aided in identifying the fungi at the species level. Identification took place at the Assiut University Mycological Centre (AUMC) in Egypt.

## Qualitative selection of L-asparaginase producing fungi

The isolated fungi were qualitatively evaluated to determine their L-asparaginase enzymatic activity through the plate assay due to its rapid and reliable screening capacity for the enzyme production (Gulati et al. 1997). The fungi were cultured on Petri dishes containing a modified Czapek’s Dox medium consisting of the following (g/L): Distilled water was used to dissolve and complete the following components: sucrose (30.0), sodium nitrate (NaNO_3_) 3.0, di-potassium hydrogen orthophosphate (K_2_HPO_4_) 1.0, potassium chloride (KCl) 0.5, magnesium sulphate (MgSO_4_.7H_2_O) 0.5, ferrous sulphate (FeSO_4_.5H_2_O) 0.001, and agar-agar 15 supplemented with phenol red as a pH indicator, with the medium adjusted to pH 6. For the control, fungi were cultivated in the medium lacking L-asparagine and supplemented with 10 g/L sodium nitrate. After incubating at 30 °C for 5 days, the development of a pink halo surrounding the fungal colonies indicated potential production of L-asparaginase, with the halo diameter measured. Triplicates were prepared for each fungal isolate.

## Quantitative evaluation of L-asparaginase activity

Fungal isolated strains capable of producing substantial levels of L-asparaginase qualitatively were chosen for further analysis under submerged fermentation conditions. They were coded, and deposited in the AUMC culture collection, Assiut, Egypt. Fifty milliliters of modified Czapek’s Dox liquid medium was inoculated with fungal isolates and incubated at 30 °C for 3 days while shaking at 100 rpm in a sterile environment, with uninoculated flasks acting as controls. After incubation, cultures were filtered and the filtrates were then subjected to centrifugation at 10,000 ×g for 20 min at 4 °C. The resultant supernatant served as the crude enzyme extract for activity assays.

The L-asparaginase activity was assessed using the method outlined by (Nafisaturrahmah et al. 2022). Estimation of the liberated ammonia during catalysis of asparagine by L-asparaginase using Nessler’s reagent was used as indication of L-asparaginase activity. The reaction mixture contained 0.5 mL of crude sample, 0.04 M L-asparagine and 0.05 M acetate buffer (pH 5.4), which was incubated for 10 min at 35 °C. Half millilitres of 1.5 M trichloroacetic acid solution was added to stop the reaction. The coupled liberated ammonia with Nessler’s reagent (MerckVR) was determined spectrophotometrically at 500 nm. The amount of L-asparaginase that caused liberation of one micromole of ammonia using the assay conditions was recorded as international unit of L-asparaginase.

## Optimization of fermentation parameters for enhanced L-asparaginase production

Four fungal isolates were chosen for further research due to their high L-asparaginase production, focusing on optimizing cultural conditions to enhance enzyme production and activity. All experiments were conducted in triplicate and data are expressed as mean ± standard deviation (SD) using one factor at a time (OFAT). Submerged fermentation was carried out in 100 mL of production medium using various carbon sources, including fructose, maltose, mannose, and starch, each at a concentration of 0.5%. The carbon source that resulted in the maximum production of the enzyme was identified for subsequent experiments. To assess the effect of nitrogen sources, the selected fungi were cultured in media supplemented with 1% concentrations of peptone, yeast extract, malt extract, and L-asparagine. All cultures were incubated at 30 °C for 72 h.

The impact of temperature on L-asparaginase production was evaluated by incubating the inoculated media at 10 °C, 30 °C, 50 °C, and 70 °C for 72 h. Similarly, the effect of pH was assessed by adjusting the media’s initial pH to 4, 6, 8, and 10, and then incubating at 30 °C for 72 h. After incubation, fungal mycelial mats were collected through filtration, and the resulting culture filtrates were utilized to assess L-asparaginase activity, following the method described by (Prasad et al. 2005).

## Partial purification of extracellular L-asparaginase enzyme

The L-asparaginase enzyme was partially purified using cold ethanol precipitation followed by a simple Diethylaminoethyl cellulose (DEAE-cellulose) ion-exchange chromatography step, as previously described for fungal L-asparaginase (Saleh et al. 2025a). After incubation, the culture broth was centrifuged (10,000 rpm, 10 min, 4 °C) to obtain the cell-free supernatant. The enzyme was partially purified using absolute ethanol (− 25 °C), followed by dissolution of the precipitated protein in Tris buffer (pH 8.0). L-asparaginase enzyme solution was dialyzed (12–14 kDa cutoff) to remove salts and low molecular weight impurities. Further purification was achieved using DEAE-cellulose ion-exchange chromatography. The column was equilibrated with phosphate buffer (100 mM, pH 8.0), and the enzyme was eluted using a stepwise sodium chloride gradient. Fractions showing the highest enzyme activity were pooled, concentrated, and stored for subsequent analyses. The partially purified enzyme was characterized by sodium dodecyl sulfate-polyacrylamide gel electrophoresis (SDS-PAGE) to analyze the molecular weight pattern of the enzyme prior to its use in downstream biological assays, including antimicrobial, antibiofilm, and cytotoxicity evaluations.

### SDS-PAGE profiling of L-asparaginase enzyme

SDS-PAGE was carried out to estimate the molecular weight of the partially purified L-asparaginase. The acrylamide gel was prepared using a 15% separating layer in 1.5 mol/L Tris-HCl buffer (pH 8.8) and a 5% stacking layer in 1.0 mol/L Tris-HCl buffer (pH 6.8). The partially purified enzyme samples were mixed with a Tris-HCl buffer containing SDS, bromophenol blue, glycerol, and β-mercaptoethanol, then heated at 90 °C for 10 min. Subsequently, 10 µL of each sample was loaded onto the gel and electrophoresed at 100 V for approximately 2 h. Upon completion, the gel was stained using Blue Silver Coomassie according to the protocol described by (Candiano et al. 2004). A wide-range molecular weight marker (Sigma Marker; 3-260 kDa) was used.

## Antimicrobial activity of L-asparaginase

### Screening for antimicrobial activity of L-asparaginase by agar well diffusion

The antimicrobial activity of the partially purified enzyme preparation was evaluated using the agar well diffusion method (Balouiri et al. 2016) against five pathogenic strains; *S. aureus* ATCC5638, *K. pneumoniae* ATCC13883, *E. coli* ATCC8379, *P. aeruginosa* ATCC27853, and *C. albicans* ATCC10231 which were obtained from Microbiological Resource Center (MIRCIN) Faculty of Agriculture, Ain Shams, University, Cairo, Egypt. Ciprofloxacin and fluconazole served as reference agents for antibacterial and antifungal activities, respectively. Microbial suspensions were prepared in Mueller-Hinton broth (MHB) and adjusted to a 0.5 McFarland standard. The final microbial suspension was adjusted to a dilution of about 10^6^ CFU/mL, which was then spread on MHA plates. Wells of 6 mm were created and filled with 20 µL of each partially purified L-asparaginase sample, with dimethyl sulfoxide (DMSO) served as a negative control, with the plates incubated at 37 °C for 24 h before measuring the inhibition zones. All experiments were performed in triplicate, and results were expressed as mean ± SD.

### Determination of minimum inhibitory concentration (MIC)

The broth microdilution method was performed to determine the MIC values of the partially purified L-asparaginase samples (Wiegand et al. 2008) in accordance with Clinical and Laboratory Standards Institute guidelines (CLSI 2024). A volume of 200 µL from each sample was dispensed into the first column of a 96-well microtiter plate, followed by serial two-fold dilutions in MHB across the rows, resulting in final concentrations ranging from 1000 to 1.9 µg/mL. Each well was then inoculated with 10 µL of standardized microbial suspension (approximately 10^6^ CFU/mL). Column 11 served as the growth control (medium with inoculum), while column 12 was used as the sterility control (medium only). Following incubation at 37 °C for 24 h, the MIC was determined as the lowest enzyme sample concentration that fully prevented visible microbial growth. Results were expressed as the mean of three independent experiments, each performed in duplicate.

### Antibiofilm activity of L-asparaginase enzyme

The antibiofilm efficacy of partially purified enzyme samples from four fungal strains against *S. aureus* ATCC5638, *K. pneumoniae* ATCC13883, *E. coli* ATCC8379, and *P. aeruginosa* ATCC27853 strains, was assessed using the crystal violet technique (Stepanović et al. 2007) at sub-MIC concentrations. Bacterial suspensions were diluted 100-fold from a 0.5 McFarland standard, followed by inoculation into 96-well microtiter plates, with DMSO used as a negative control. Following 24-hour incubation at 37 °C, the wells were washed using phosphate-buffered saline (PBS). Biofilms were fixed with 99% methanol and subsequently stained with 0.5% crystal violet, with any excess stain removed afterward. The stained biofilm was then solubilized in 95% ethyl alcohol, and the absorbance was measured at 570 nm. Each assay was performed in triplicate across three independent experiments, and results were expressed as mean values. The percentage of biofilm inhibition was calculated according to the following formula:

$$ {\mathrm{Percentage}}(\% ){\mathrm{inhibition}} = \left[ {\frac{{\left( {{\mathrm{OD}}_{{{\text{negative control}}}} - {\mathrm{OD}}_{{{\mathrm{experimental}}}} } \right)}}{{{\mathrm{OD}}_{{{\text{negative control}}}} }}} \right] \times {\mathbf{100}}. $$

### Determination of cell viability and cytotoxicity using the MTT assay

Human hepatocellular carcinoma (HePG2) and breast cancer (MCF-7) cell lines were acquired from the Cell Culture Laboratory, National Research Centre, Giza, Egypt. The cells were cultured in Dulbecco’s Modified Eagle Medium (DMEM) supplemented with 10% fetal bovine serum and 100 U/mL penicillin, under a humidified environment containing 5% CO_2_ at 37 °C. To assess the cytotoxic potential of the enzyme, the MTT assay was performed following the method of (Mosmann 1983). Briefly, 100 µL of the cell suspension (1 × 10^5^cells/mL) was seeded into 96-well microplates and incubated for 24 h to allow monolayer formation. Subsequently, varying concentrations of the partially purified enzyme (31.25, 62.5, 125, 250, 500, and 1000 µg/mL) were added to the wells. The plates were then incubated again for 24 h at 37 °C in a 5% CO_2_ environment. After incubation, the medium was discarded and replaced with MTT solution to a final concentration of 0.5 mg/mL. The plates were incubated for an additional 3 h to allow the formation of formazan crystals, which were then solubilized by adding 100 µL of DMSO. Absorbance was measured at 570 nm with a reference wavelength of 630 nm using an ELISA microplate reader (ELX800, BioTek). The IC_50_ values were graphically calculated. All assays were conducted in triplicate to ensure reproducibility.

Cytotoxic effects were further validated through microscopic examination. Both treated and untreated (control) cell lines were observed for morphological alterations, following the procedure described by (Bhat and Marar 2015).

### Computational study

Molecular docking and molecular dynamics simulations were used to supplement experimental results by offering structural information about the interactions between the enzyme and the substrate.

### Homology modelling

Since the crystallographic structure of *A. nidulans* L-asparaginase is not available in the Protein Data Bank (PDB), the homology model was constructed by searching for a template. To build a homology model of *A. nidulans* L-asparaginase, the amino acid sequence of the L-asparaginase protein was obtained from the website http://www.uniprot.org) (accession No.: Q5BGN0) (Bateman et al. 2025). The basic local alignment search tool BLAST (http://www.ncbi.nim.nih.gov/blast.cgi*)* (Camacho et al. 2009) was used to identify the protein with the most sequence similarity to L-asparaginase from PDB. BLAST showed that the appropriate template for *A. nidulans* L-asparaginase with 52.55% similarity was Chain A, *Erwinia carotovora* L-asparaginase (PDB ID: 2JK0).

### Binding site prediction and docking analysis

The COACH meta-server approach (https://zhanggroup.org/COACH/) (Yang et al. 2013) was utilized for predicting protein-ligand binding sites. Subsequently, molecular docking analyses were conducted for L-asparagine (L-Asn). Input files for the identified chemical compounds and protein structures were generated using AutoDockTools (Morris et al. 2009), while OpenBabel v2.4 (O’Boyle et al. 2011) was employed to preprocess all ligands. Molecular docking was performed using `AutoDock Vina (Jaghoori et al. 2016), with a grid box corroborated the COACH for the protein docking prediction (C-score = 0.96). Finally, interactions between the docked compounds and key proteins were analyzed and visualized using Pymol and Discovery Studio Visualizer 21.1.0.2. The initial structures for the simulations were derived from the predicted binding orientations of the complexes obtained via molecular docking.

### Molecular dynamics study

The initial structures for the simulations were derived from the predicted binding orientations of the complexes obtained via molecular docking. Prior to simulation, ligand and protein structures were refined and, if necessary, displaced using CHARMM-GUI to optimize initial configurations and ensure proper system preparation (Jo et al. 2008).

Molecular dynamics (MD) simulations were carried out utilizing the GROMACS 2020.2 software package (Abraham et al. 2015). The simulations employed the AMBER99SB-ILDN force field (Lindorff-Larsen et al. 2010). Given that this force field lacks parameters for the ligands, ANTECHAMBER was used to parameterize all ligands to ensure compatibility with the General Amber Force Field (GAFF) (Wang et al. 2004). Charges were assigned using the AM1-BCC method, and ACPYPE (Sousa da Silva, Vranken 2012) was used to transform the ligand topologies into a format suitable for GROMACS.

All simulations were performed in an explicit water environment, utilizing the TIP3P model. The complexes were placed in a dodecahedral box system and neutralized by incorporating Na⁺ and Cl⁻ ions (Mark and Nilsson 2001). Energy minimization was carried out utilizing the steepest descent method, with a maximum force threshold (Fmax) set to 1000 kJ mol^-1^ nm^-1^. The systems were equilibrated for 200 ps each using the NVT and NPT ensembles, followed by a production run lasting 25 ns. The temperature was kept at 300 K employing the V-rescale algorithm (Rangan et al. 2018), while the Parrinello-Rahman barostat was used to control the system pressure (Parrinello and Rahman 1981).

The LINCS (LINear Constraint Solver) algorithm (Hess et al. 1997) was employed to maintain bond lengths. Long-range electrostatic interactions were addressed with the Particle Mesh Ewald (PME) technique (Darden et al. 1993), using a timestep of 2 fs for all simulations. The cutoff distance was established at 1 nm.

### Statistical analysis

Statistical analysis was conducted using one-way analysis of variance (ANOVA) to evaluate differences among groups.

## Results

### Isolation and identification of fungi from soil and sewage samples

A total of fourteen fungal isolates were recovered from wastewater and soil samples. Macroscopic and microscopic examinations confirmed that all isolates were filamentous fungi. Based on their morphological characteristics and with reference to standard identification manuals, the fungi were identified to the species level as follows: *A. flavus* var. *columinaris* (2 isolates), *A. flavus* var. *flavus* (3 isolates), *A. niger* (3 isolates), *A. terreus* (3 isolates), and one isolate each of *A. nidulans*, *Penicillium purpurogenum*, and *Rhizopus stolonifer*, as presented in Table 1.

### Assessment of L-asparaginase production

**Fig. 1:**
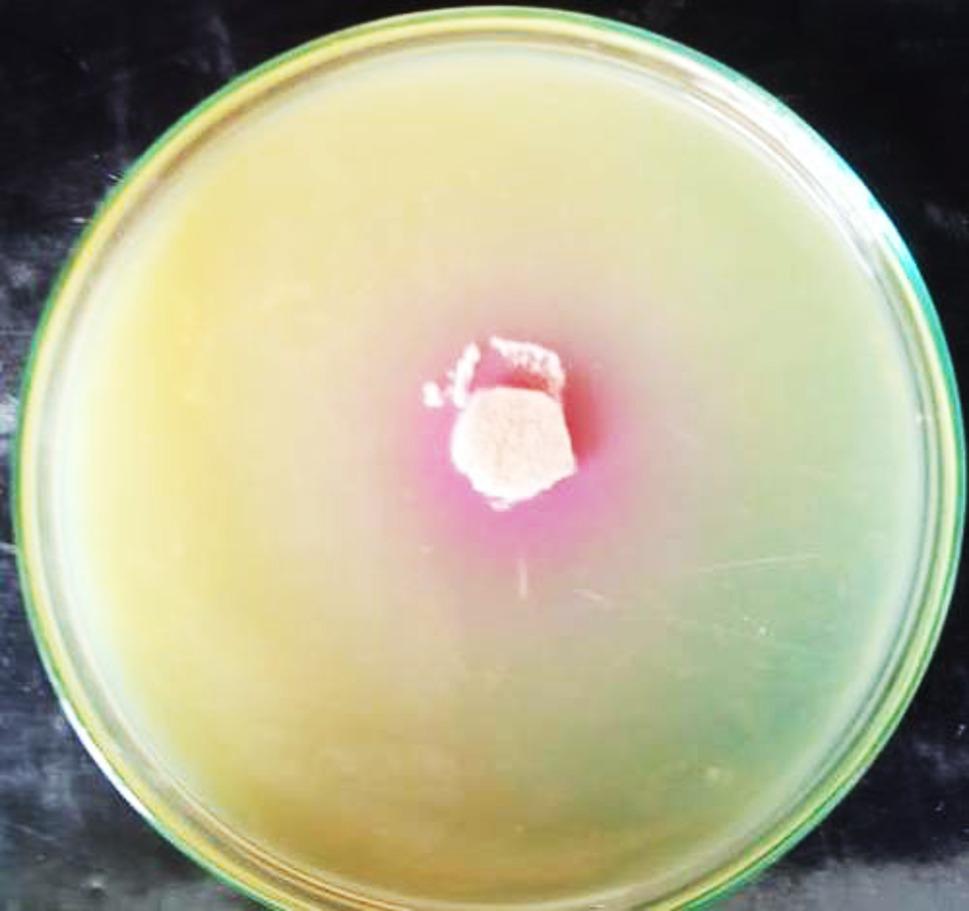
Preliminary qualitative screening for L-asparaginase production by *A. terreus* (isolate 2) on modified Czapek’s Dox agar supplemented with phenol red. The formation pink halo around the fungal culture indicates extracellular L-asparaginase activity due to ammonia release during L-asparagine hydrolysis

Screening for the enzyme production was carried out by the plate method, which is a quick and qualitative technique for detecting enzyme synthesis. The pink zone surrounding colonies was used as an indicator of positive strains, as microbial activity hydrolyzes the enzyme into L-aspartic acid and ammonia, changing the medium colour from yellow (acid) to pink (alkaline). Ten strains exhibited positive outcomes for the enzyme production. Among them, four individual isolates, *A. nidulans*, *A. flavus* var. *flavus* isolate 3, *A. flavus* var. *flavus* isolate 2, and *A. terreus* isolate 3, exhibited the highest activity, with zone diameters of 40 ± 0.03, 24.2 ± 0.12, 21.4 ± 0.1, and 20 ± 0.08 mm, respectively, as shown in Table 1; Fig. 1. The authenticated four isolates were coded as AUMC17371, AUMC17373, AUMC17374, AUMC17372 respectively.

**Table 1:** Fungal isolates, their sources, and the diameters of L-asparaginase production zones

Fungal isolates		Source	Pink zone diameter (mm)
1	A. flavus var. columinaris (1)	Soil	14.2 ± 0.03
2	A. *flavus* var. *columinaris* (2)	Sewage	14.2 ± 0.07
3	*A. flavus* var. *flavus* (1)	Sewage	17.1 ± 0.05
4	*A. flavus* var. *flavus* (2) [AUMC17374]	Sewage	21.4 ± 0.1
5	*A. flavus* var. *flavus* (3) [AUMC17373]	Soil	24.2 ± 0.12
6	*A. niger* (1)	Soil	–
7	*A. niger* (2)	Sewage	–
8	*A. niger* (3)	Sewage	–
9	*A. nidulans* [AUMC17371]	Soil	40 ± 0.03
10	*A. terreus* (1)	Sewage	14 ± 0.06
11	*A. terreus* (2)	Sewage	16 ± 0.02
12	*A. terreus* (3) [AUMC17372]	Soil	20 ± 0.08
13	*Rhizopus stolonifer*	Soil	–
14	*Penicillium purpurogenum*	Sewage	18 ± 0.09

Values are presented as mean value ± SD

The quantitative assay of the crude extracts from the four fungal isolates with the highest L-asparaginase production confirmed the results obtained from the plate-based qualitative screening. Among these isolates, *A. nidulans* AUMC17371 exhibited the highest enzyme activity (1.35 ± 0.02 U/mL) (*p* < 0.05), followed by *A. flavus* AUMC17373 (1.25 ± 0.05 U/mL). In contrast, *A. flavus* AUMC17374 and *A. terreus* AUMC17372 showed comparatively lower activities of 0.97 ± 0.01 U/mL and 0.79 ± 0.05 U/mL, respectively. These results confirm the results obtained by the preliminary screening method.

### Optimization of culture conditions for enhanced L-asparaginase production

**Fig. 2:**
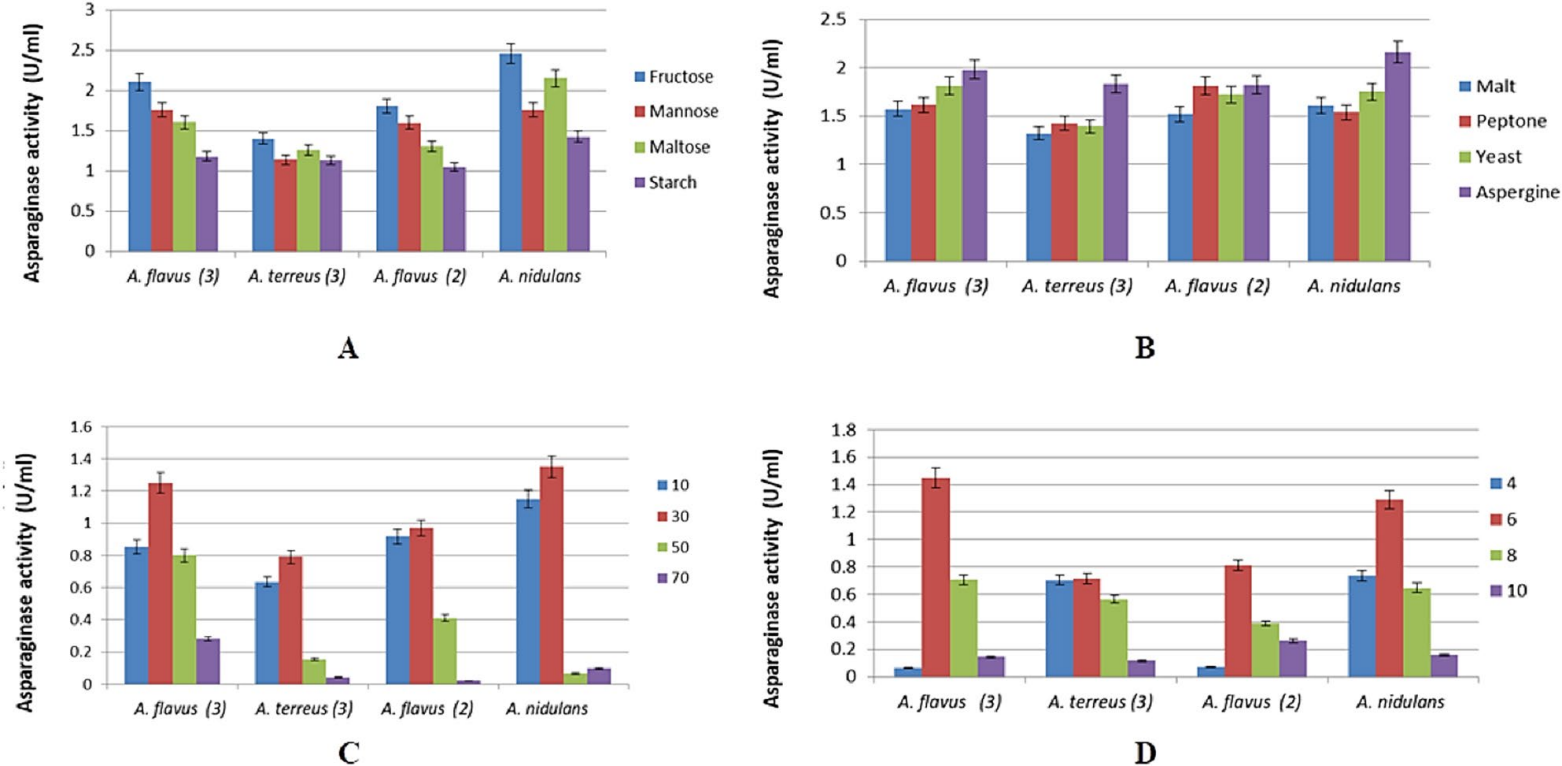
Optimization of culture conditions for L-asparaginase production by the four fungal strains under submerged fermentation. The effects of various (**A**) carbon sources (**B**) nitrogen sources (**C**) incubation temperatures (**D**) initial pH values on enzyme activity were evaluated. Results were presented as mean ± SD (*p* < 0.05)

The impact of varied composition and cultivation conditions on L-asparaginase synthesis by *A. nidulans* AUMC17371, *A. flavus* AUMC17373, *A. flavus* AUMC17374, and *A. terreus* AUMC17372 was investigated. In general, carbohydrates serve as carbon sources during fermentation processes. The examination of various carbon sources on L-asparaginase synthesis indicated that fructose, a monosaccharide, exhibited the greatest enzyme activity, whereas starch resulted in the lowest enzyme production, as illustrated in Fig. 2A. Among the nitrogen sources examined, asparagine in the medium enhanced fungal growth and subsequently promoted the enzyme production. Peptone led to the lowest enzyme production from *A. flavus* AUMC17373, whereas malt resulted in the least enzyme production from the other strains, as depicted in Fig. 2B.

Peak enzyme production was obtained at 30 °C, with a gradual decline observed as the incubation temperature rose (Fig. 2C). To identify the optimal pH for maximum enzyme output, the effect of initial pH was analyzed. The enzyme activity reached its highest point at pH 6, followed by a decrease at pH 8 and pH 10, as shown in Fig. 2D.

#### Characterization of L-asparginase using SDS - PAGE

**Fig. 3:**
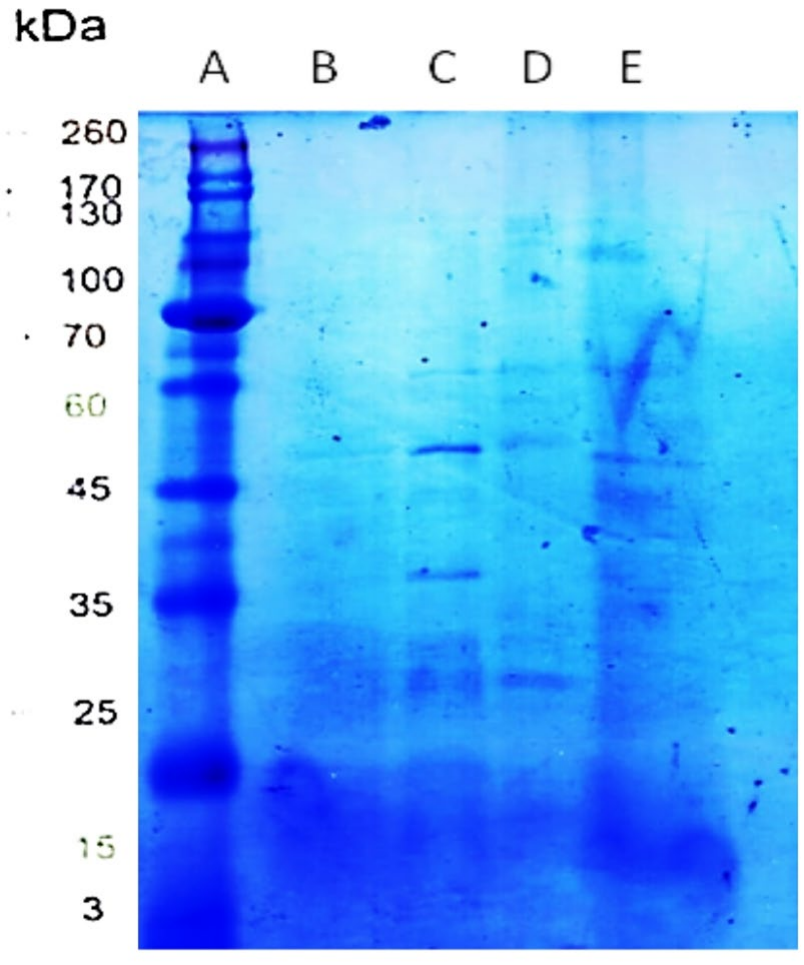
SDS-PAGE analysis of partially purified L-asparaginase. **A** Molecular marker (3 kDa – 260 kDa). **B** *A. flavus* AUMC17373. **C** *A. flavus* AUMC17374. **D** *A. terreus* AUMC17372. **E** *A. nidulans* AUMC17371

SDS-PAGE analysis was performed to estimate the production of the enzyme by *A. nidulans* AUMC17371, *A. flavus* AUMC17373, *A. flavus* AUMC17374, and *A. terreus* AUMC17372, and to assess its molecular weight. Protein bands corresponding to molecular weights ranging from 35 to 45 kDa were observed in. The presence of clear bands in the expected molecular weight range with the quantitative analysis results supports the synthesis of L-asparaginase by these fungal isolates (Fig. 3).

### Antimicrobial assays of L-asparaginase enzyme

The partially purified preparations of *A. flavus* AUMC17374, *A. flavus* AUMC17373, *A. nidulans* AUMC17371, and *A. terreus* AUMC17372 were preliminarily evaluated for antimicrobial activity using the cup-plate method. The inhibition zone diameters (mm) were recorded against five microbial isolates. The results shown in Table 2 showed that all preparations exhibited variable antimicrobial activity. The strongest antagonistic activity was observed against *S. aureus* ATCC5638 and *P. aeruginosa* ATCC27853, while the weakest effect was recorded against *E. coli* ATCC8379. Among the tested fungi, *A. nidulans* AUMC17371 exhibited the highest activity against *S. aureus* ATCC5638 (24 ± 0.28 mm), followed by *A. flavus* AUMC17373 against *P. aeruginosa* ATCC 27,853 (23 ± 0.45 mm). In contrast, all preparations displayed low antifungal activity against *C. albicans* ATCC10231, with inhibition zones ranging from 10 ± 0.2 to 14 ± 0.36 mm.

**Table 2:** Antimicrobial activity of enzyme preparations against pathogenic strains by the cup-plate method

	Diameter of zone of inhibition (mm)				
	*S. aureus* ATCC5638	*E. coli* ATCC8379	*K. pneumoniae* ATCC13883	*P*. *aeruginosa* ATCC27853	*C. albicans* ATCC10231
*A. flavus* AUMC17374	20 ± 0.30	13 ± 0.60	14 ± 0.45	16 ± 0.57	14 ± 0.36
*A. flavus* AUMC17373	19 ± 0.25	10 ± 0.50	15 ± 0.72	23 ± 0.45	13 ± 0.30
*A. nidulans* AUMC17371	24 ± 0.28	12 ± 0.80	12 ± 0.68	18 ± 0.25	13 ± 0.44
*A. terreus* AUMC17372	17 ± 0.60	11 ± 0.59	16 ± 0.20	17 ± 0.18	10 ± 0.20

Data is represented as mean ± SD

The minimum inhibitory concentration (MIC) values of the four enzyme preparations were assessed using the broth microdilution method. MIC results shown in Table 3, revealed that among all tested preparations, *A. nidulans* AUMC17371 exhibited the most potent antimicrobial effect, recording the lowest MIC value (31.25 µg/mL) against *S. aureus* ATCC5638 followed by *A. flavus* AUMC17373 and AUMC17374 (MIC = 62.5 µg/mL for both). *A. flavus* AUMC17373 also showed moderate activity against *P. aeruginosa* ATCC27853 (62.5 µg/mL). In contrast, the preparations demonstrated weak activity against *E. coli* ATCC8379 and *C. albicans* ATCC10231.

**Table 3:** MIC values (µg/mL) of enzyme preparations against pathogenic strains

	MIC (µg/mL)				
	*S. aureus* ATCC5638	*E. coli *ATCC8379	*K. pneumoniae* ATCC13883	*P*. *aeruginosa* ATCC27853	*C.* *albicans* ATCC10231
*A. flavus* AUMC17374	62.5	1000	500	250	500
*A. flavus* AUMC17373	62.5	> 1000	125	62.5	500
*A. nidulans* AUMC17371	31.25	500	125	125	500
*A. terreus* AUMC17372	125	> 1000	125	125	> 1000

#### Inhibition of biofilm formation by L-asparaginase preparations

The antibiofilm potential of the four enzyme preparations were evaluated against the four biofilm-forming strains by measuring biofilm inhibition percentages at sub-inhibitory concentrations. Results showed that all the tested preparations exhibited biofilm inhibition against the tested strains. *A. flavus* AUMC17373 enzyme preparation displayed the strongest biofilm inhibition nearly against all tested strains. *A. terreus* AUMC17372 showed the lowest percentage of biofilm inhibition (22%) against *K. pneumoniae* ATCC13883 biofilm, as shown in Table 4. The inhibition percentages ranged from 47 to 69% for Gram-positive *S. aureus* ATCC5638 and 22–61% for Gram negative tested strains.

**Table 4:** Biofilm Inhibition percentages of enzyme preparations against pathogenic strains

	% Inhibition of the biofilm			
	*S. aureus* ATCC5638	*E. coli* ATCC8379	*K. pneumoniae* ATCC13883	*P*. *aeruginosa* ATCC27853
*A. flavus* AUMC17374	60 ± 1.2	57 ± 0.9	52 ± 5.2	36 ± 6.1
*A. flavus* AUMC17373	69 ± 1.5	54 ± 4.6	61 ± 3.5	44 ± 4.3
*A. nidulans* AUMC17371	65 ± 3.4	30 ± 2.8	46 ± 2.1	25 ± 8.2
*A. terreus* AUMC17372	47 ± 2.7	41 ± 4.1	22 ± 7.9	30 ± 0.9

Data is represented as mean ± SD of three independent experiments and percentages were calculated in relation to the untreated control

#### Anticancer activity of *A. flavus* AUMC17374 enzyme preparations on MCF-7 and HepG2

**Fig. 4:**
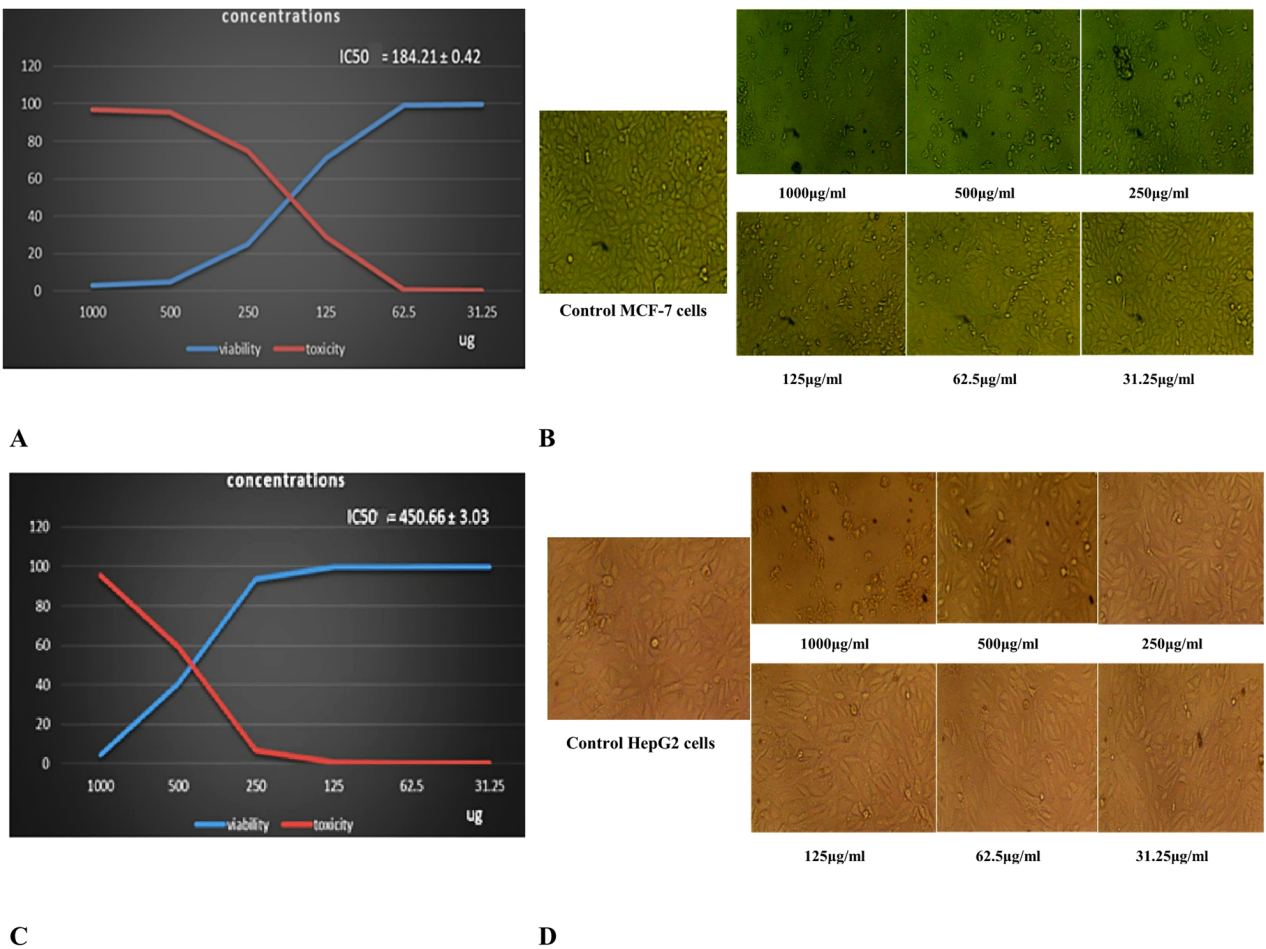
Effect of *A. flavus* AUMC17374 L-asparaginase on the viability and cytotoxicity of MCF-7 and HepG2 cells at different concentrations.** A**,** B** MCF-7 cells;** C**,** D** HepG2 cells. Values are presented as mean ± SD

The cytotoxic effects of the L-asparaginase enzyme from *A. flavus* AUMC17374 strain on MCF-7 and HepG2 cell lines were evaluated using the MTT metabolic assay. This investigation evaluated the cytotoxic effect of different concentrations of the enzyme from 31.25 to 1000 µg/mL, with untreated cell lines utilized as the negative control. The findings indicated a concentration-dependent reduction in cell viability, indicating that the two cell lines exhibited different sensitivities to the partially purified enzyme, as shown in Fig. 4A and C. Cytotoxicity was induced in MCF-7 cells, yielding an IC_50_ value of 184.21 µg/mL, whereas HepG2 cells showed a higher IC_50_ value of 450.66 µg/mL. Figure 4A and C illustrate the percentage cell viability at different enzyme concentrations (µg/mL).

Furthermore, morphological changes increased with rising concentrations of the *A. flavus* AUMC17374 L-asparaginase. Apoptotic features were observed in both MCF-7 and HepG2 cells, as shown in Fig. 4B and D, respectively. In contrast, no noticeable morphological alterations were detected in the untreated control cells. Figure 4B and D display the morphological changes in MCF-7 and HepG2 cells compared to the untreated controls.

#### Computational study

Among the isolated fungal strains *A. nidulans* AUMC17371 was selected for computational studies since it exhibited the maximum production level of L-asparaginase, in addition to showing strong antimicrobial activity.

#### The binding site and molecular docking analysis

**Fig. 5:**
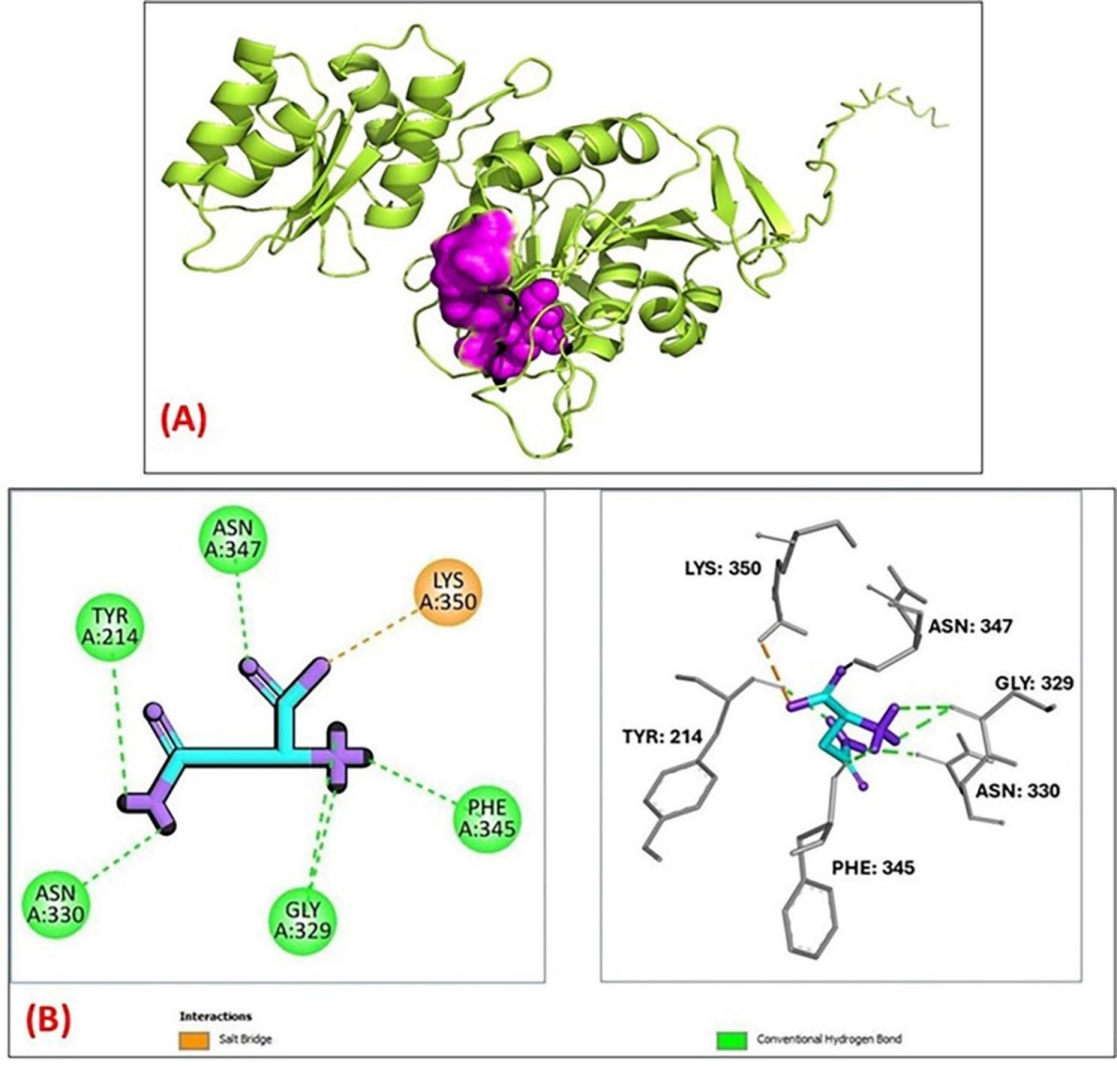
**A** 3D *A. nidulans* AUMC17371 L-asparaginase protein predicted (lemon color) and their binding site residues (magenta color) according to COACH analysis, **B** 2D & 3D Views of L-asparagine docked in L-asparaginase enzyme predicted active site

According to the COACH analysis, the active site of the protein comprises amino acids at positions 62, 63, 109, 110, 111, 143, 144, 145, and 169, with a confidence score (C-score) of 0.96 as shown in Fig. 5A. This predicted binding site was subsequently used to assess the binding affinity of L-asparagine (L-Asn).

The molecular docking analysis of L-Asn with *A. nidulans* L-asparaginase reveals critical binding interactions that enhance the enzyme’s substrate specificity and catalytic efficiency. L-Asn interacted with the active site of L-asparaginase enzyme (Docking Score= − 4.67 kcal/mol) via conventional five hydrogen bonds are observed between L-Asn and Asn347 (backbone amide), Tyr214 (hydroxyl group), and Asn330 (side chain carbonyl), facilitating precise positioning for nucleophilic attack during hydrolysis as well as presence of both Gly329 and Phe345 suggests conformational flexibility in the binding site. A prominent salt bridge forms between the carboxylate group of L-Asn and the positively charged side chain of Lys350, which is essential for proper orientation of the substrate as displayed in Fig. 5B.

### Molecular dynamic study

**Fig. 6:**
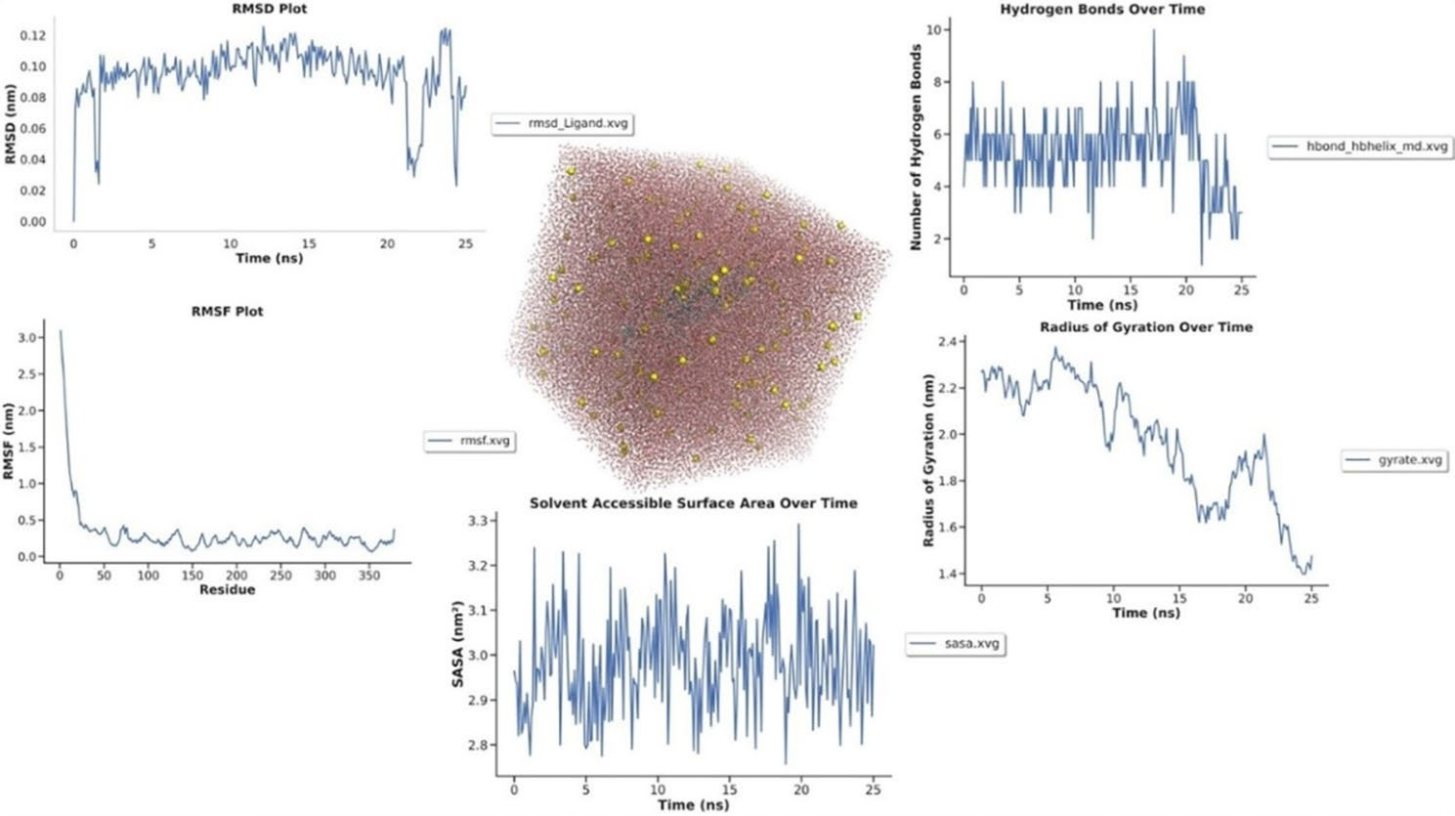
Molecular dynamics study of L-asparagine in the L-asparaginase enzyme’s putative active site over a 25 ns simulation period

Molecular dynamics (MD) simulations offer important information about the structure and dynamical behavior of biomolecular interactions. Over a 25-nanosecond simulation period, the interaction between L-Asn and the L-Asparaginase enzyme was analyzed using multiple MD descriptors, including hydrogen bonding, solvent accessibility, structural compactness, ligand stability, total energy, and residue-specific flexibility via root mean square fluctuation (RMSF) as shown in Fig. 6.

The hydrogen bonding analysis revealed an average of approximately 5 hydrogen bonds between L-Asn and the enzyme throughout the simulation. This consistent interaction suggests a stable binding mode that may contribute to effective substrate recognition and enzymatic catalysis. The helical content analysis further indicated that the secondary structure of L-asparaginase remains largely stable, reinforcing the integrity of the active site. The solvent-accessible surface area (SASA) analysis showed a mean value of ~ 3.0 nm², indicating moderate solvent exposure of the enzyme-ligand complex. This suggests that L-Asn binding does not significantly disrupt the overall solvation dynamics of the enzyme. Additionally, the radius of gyration (Rg) remained stable, with an average value of approximately 2.45 nm, suggesting that the enzyme maintains its compact structure throughout the simulation. The relatively low variation in Rg values supports the notion that L-Asn binding does not induce significant conformational changes.

The ligand’s root mean square deviation (RMSD) showed minimal fluctuations, with values close to 0 throughout the simulation. This finding suggests that L-Asn remains tightly bound within the enzyme’s active site, further supporting its stable interaction and limited mobility. Furthermore, the total energy analysis revealed a consistently negative energy profile, ranging from approximately − 2,518,850 to − 2547, 052 kJ/mol. This stable energy trend confirms that the L-Asn -L-asparaginase complex remains in a well-equilibrated state throughout the simulation, reinforcing the stability of the binding interaction.

The RMSF analysis provided additional insights into the local dynamics of the enzyme. The majority of residues exhibited very low fluctuations (RMSF < 0.001 nm), indicating high rigidity, particularly in regions critical for catalytic function. A few residues (e.g., 2, 5, 7, 12, 278, and 282) displayed slightly higher fluctuations (up to ~ 0.0007 nm), likely corresponding to flexible loop regions or peripheral residues. Importantly, residues near the active site maintained minimal fluctuations, aligning with the observed stability in hydrogen bonding and ligand RMSD. This suggests that while the enzyme retains overall structural rigidity, minor flexibility in non-catalytic regions may facilitate substrate accessibility without compromising active-site stability. The strong binding affinity and stable interaction observed in molecular docking and dynamics simulations correlate with the experimentally measured high enzymatic activity of *A. nidulans* AUMC17371 (1.35 ± 0.02 U/mL), suggesting a structural basis for its superior catalytic performance.

## Discussion

Natural products, particularly those from microbial origins, continue to be vital in the development of novel therapeutics, especially in oncology and infectious disease treatment (Niazi et al. 2023). Among microbial sources, fungi have been widely recognized for their capacity to produce a wide range of bioactive metabolites, including enzymes with antimicrobial and anticancer properties (Emmanuel and Igoche 2022).

In this study, soil and sewage wastewater samples were selected as sampling sources. Soil and sewage habitats are particularly rich in microbial diversity, particularly *Aspergillus* species and are known to harbour a variety of fungi capable of producing biologically active compounds. In fact, over 60% of known antimicrobial compounds have been isolated from soil microbes (Niazi et al. 2023). Fourteen fungal isolates from sewage and soil samples were screened for L-asparaginase production. Ten strains showed positive results in plate assays, with *A.nidulans* AUMC17371, *A. flavus* AUMC17373, *A. flavus* AUMC17374, and *A. terreus* AUMC17372 demonstrating the highest enzyme production. Quantitative analysis confirmed that *A. nidulans* AUMC17371 exhibiting highest L-asparaginase activity.

Numerous studies have reported effective production of L-asparaginase by various fungal and bacterial isolates obtained from different environmental niches. *E. coli* isolates from sewage (Borah, 2012) and *P. aeruginosa* strains from garden soil (Fatima, 2019) have been recognized as promising producers of L-asparaginase. Additionally, Priya and Subhashini (2022) proved that fungi such as *Rhizopus* spp. from fruit peel-degraded soil, *Aspergillus* spp. from vegetable waste soil, and *Aspergillus lentulus* from *Annona muricata* have shown high enzyme activity, revealing significant variation in enzyme production across species and strains, highlighting the importance of selecting the most efficient isolates for further optimization and potential medical applications (Chinnadurai and Govindasamy 2024).

Vimal and Kumar (2017) highlighted that higher enzymatic activity correlates with greater enzyme production potential, which aligns with our observations of large pink zones. AdditionallyAl-Bedak et al. (2025); Saleh et al. (2025b) confirmed that various fungal species, including *Fusarium* sp., *Penicillium* sp., and *Aspergillus* sp., exhibited L-asparaginase activity, supporting the idea that multiple fungal strains may have the ability to manufacture this enzyme.

Optimization of extraction conditions is a crucial step in maximizing the recovery of desired bioactive metabolites from natural sources (Ganesan et al. 2018). The current study investigated the effect of various media compositions and culture conditions on L-asparaginase production by the four selected fungal strains. Of the carbon sources evaluated, fructose led to the greatest enzyme activity, whereas starch resulted in the lowest yield. Our results align with previous reports indicating that glucose, lactose, sucrose, and maltose can enhance L-asparaginase production depending on the fungal species (Sanjotha 2017; da Cunha et al. 2022). Our study demonstrates that the best nitrogen source for increasing the production of L-asparaginase is asparagine, while peptone and malt led to the lowest enzyme production in various fungal strains. This is consistent with the results obtained by Sisay et al. (2024), who demonstrated enhanced enzyme production when L-asparagine provided as the sole nitrogen source. Furthermore, Elshafei and El-Ghonemy (2015) and da Cunha et al. (2022) reported that casein and peptone were effective nitrogen sources for the enzyme production, further supporting the importance of organic nitrogen sources in optimizing enzyme production.

In the present work, maximum enzyme production was obtained at an incubation temperature of 30 °C, with a gradual decline observed as the temperature increased. These results align with Chinnadurai and Govindasamy (2024), whereas Sanjotha (2017) reported maximum activity at 35 °C. Temperature has a crucial role in microbial growth and metabolism. Lowering the ambient temperature of growth conditions diminishes enzyme synthesis and metabolic activity (Belhadj Slimen et al. 2016). However, excessively high temperatures can cause destruction of the enzyme (Manzoni et al. 2012). Furthermore, pH plays a significant role in L-asparaginase production, with optimal enzyme activity at pH 6, which is consistent with the finding of Chinnadurai and Govindasamy (2024) reporting that slightly acidic pH values are conducive to enzyme production. On the other hand Sanjotha (2017) reported the highest enzyme production at pH 7.5, indicating that different fungal strains may have varying pH optima for the synthesis of L-asparaginase.

SDS-PAGE analysis was carried out in our study to estimate the molecular weight of L-asparaginase produced by the selected fungal strains. The results revealed that the enzyme was obtained in a partially purified form, evidenced by the appearance of protein bands with a molecular weight ranging between 35 and 45 kDa. Likewise, Dutta et al. (2015) demonstrated that the enzyme produced by *A. fumigatus* exhibited a molecular weight of approximately 35 kDa. On the other hand, L-asparaginase has been reported to exhibit varying molecular weights based on the microbial origin. Vala et al. (2018) was reported that *A. niger* generated 90 kDa of L-asparaginase, whereas El-Naggar et al. (2016) confirmed the purification of the enzyme, exhibiting a molecular weight of 53 kDa for the *Streptomyces fradiae* enzyme. Fernandes et al. (2021) discovered that *A. caespitosus* produced two L-asparaginase protein bands of 97 kDa and 45 kDa.

Natural products, particularly those derived from fungi and bacteria, have shown great potential as sources of bioactive compounds with strong antimicrobial activity (Hayashi et al. 2013). In this study, the four L-asparaginase most producers fungal extracts were partially purified and were evaluated for antimicrobial activity. All enzyme preparations exhibited varying degrees of antimicrobial activity, with the highest effects observed against *P. aeruginosa* and *S. aureus* strains. MIC analysis revealed that *A.nidulans* showed the highest potency against *S. aureus*, while *A. flavus* AUMC17373 exhibited strong activity against *P. aeruginosa*. In consistent with current results, Raj et al. (2016) highlighted the enzyme’s antimicrobial efficacy, paving the way for its application in infection control. Vimal and Kumar (2017) research further confirmed the antimicrobial potency of L-asparaginase against *S.aureus*,* Proteus vulgaris*,* P. aeruginosa*,* Listeria monocytogenes*, and *Salmonella typhimurium*. An in silico study by Vimal and Kumar (2021) reported that the antimicrobial effects were further enhanced by encapsulating L-asparaginase within chitosan nanoparticles, improving its stability and delivery.

Biofilm-associated multidrug resistance (MDR) represents a significant challenge in hospital-acquired infections, contributing to increased morbidity and mortality rates, while also imposing substantial financial burdens through increased healthcare costs and extended hospital stays (Assefa and Amare 2022). In the present work *A. flavus* AUMC17373 L-asparaginase preparation displayed the strongest biofilm inhibition nearly against all tested strains ranging from 44% to 69% biofilm inhibition. The observed reduction in biofilm formation suggests that partially purified L-asparaginase may interfere with biofilm development under experimental conditions, supporting further investigation into their antibiofilm mechanisms.

These current results are consistent with the findings of Muslim et al. (2016), which demonstrated that L-asparaginase possesses significant antibiofilm effect against various pathogenic bacteria capable of forming biofilms. Notably, the enzyme showed the highest antibiofilm effect against *K. pneumoniae*, reducing biofilm formation by 32%, followed by *P. aeruginosa* with a 41% reduction, in comparison to the control group, which exhibited 100% biofilm formation. Further evidence was provided by Upadhyay et al. (2024), who suggested that L-asparaginase not only inhibits biofilm formation but also interferes with key proteins responsible for maintaining biofilm structure, particularly in *Salmonella Typhi*. Their study combined in vitro assays with computational modeling, revealing that L-asparaginase interacts with biofilm-associated proteins. Protein–protein docking analyses showed high binding affinities, with the strongest interaction observed between L-asparaginase and BcsA (− 219.8 kcal/mol), a protein critical for biofilm integrity.

In the present study, various concentrations of partially purified L-asparaginase from *A. flavus* AUMC17374 strain against MCF-7 and HepG2 cell lines was examined through the MTT assay. The enzyme preparation reduced breast cancer and hepatocellular carcinoma cell viability, showing IC_50_ values of 184.21 and 450.66 µg/µL, respectively. There are many previous studies have been shown the fungal L-asparaginase enzyme suppress a number of cancer cell lines, including MCF-7, 2 A-549, HCT-116, and HepG (Benchamin et al. 2019; El-Gendy et al. 2017, 2018). Further examination revealed that the *A. flavus* AUMC17374 enzyme promotes apoptosis in breast cancer cell lines. Apoptosis refers to a controlled and regulated process of cellular self-destruction which is considered the most desirable mechanism of tumor cytotoxicity in cancer therapies (Elmore 2007). According to Saleh et al. (2025a) the treatment of MCF-7 breast cancer cells with purified enzyme derived from a *Bacillus strain*, at concentrations between 31.25 and 1000 µg/mL, resulted in marked cytotoxicity, exhibiting IC_50_ value of 49.3 µg/mL. Similarly, da Silva Duarte et al. (2024) proved that L-asparaginase derived from *A. niger* exhibited significant cytotoxic effects against HeLa (cervical cancer) cells. These findings support the potential of microbial derived L-asparaginase as a promising candidate for pharmaceutical applications. Its favourable properties such as enzymatic activity, stability, and relatively low cytotoxicity towards non-cancerous cells suggest its suitability as an alternative agent in cancer treatments, potentially overcoming some of the drawbacks associated with traditional treatments (Saleh et al. 2025a).

Computational analysis was conducted in our study, as molecular docking confirmed stable binding of L-asparagine to *A. nidulans* AUMC17371 L-asparaginase (− 4.67 kcal/mol) through hydrogen bonding and a salt bridge with Lys350. Molecular dynamics simulations over 25 ns indicated structural stability of the enzyme–substrate complex, with consistent hydrogen bonding, stable RMSD, compact Rg, and minimal active-site fluctuations. As stated by Macalino et al. (2015), computational drug discovery tools play a pivotal role in identifying and optimizing potential therapeutic molecules. These methods are crucial for refining the extensive chemical space, allowing researchers to concentrate on the most promising candidates for further study. By predicting key properties such as pharmacokinetics and pharmacodynamics, these tools provide insights into the behaviour of molecules within biological systems, which can be later validated through experimental studies. Additionally, they offer a deeper understanding of how molecules interact with their target proteins or enzymes, including mechanisms of binding, electron transfer, and the regulation of enzymatic activity. This information is crucial for designing tailored treatment approaches to effectively combat specific diseases.

Although this study demonstrates promising antimicrobial, antibiofilm, and cytotoxic activities of partially purified fungal L-asparaginase, it has certain limitations. The enzyme was only partially purified, and full biochemical characterization was not performed. Production optimization focused on the most influential factors as carbon source, nitrogen source, temperature, and pH, while other parameters were not explored. In addition, the use of a partially purified enzyme in cytotoxicity assays represents a limitation, as the presence of residual fungal proteins or metabolites may contribute to or interfere with the observed cytotoxic effects, potentially leading to an overestimation or misinterpretation of the enzyme’s true anticancer activity. Full purification of L-asparaginase would allow a more accurate evaluation of its intrinsic cytotoxic potential, specificity, stability, and dose–response behavior, as well as its safety profile. Future research should aim for complete purification and detailed biochemical characterization, as well as expanded optimization including additional culture conditions and fermentation strategies. In vivo studies and protein engineering approaches could further enhance catalytic efficiency, reduce immunogenicity, and improve the therapeutic applicability of fungal L-asparaginase. Such studies are essential for promoting fungal L-asparaginase as a potential therapeutic candidate for treating infectious diseases and cancer.

## Conclusion

In this research, L-asparaginase from fungal isolates obtained from soil and sewage was successfully produced and optimized. The partially purified L-asparaginase from the four *Aspergillus* isolates with the highest enzyme levels exhibited significant antimicrobial and antibiofilm activities against clinically relevant pathogens. Furthermore, *A. flavus* AUMC17374 partially purified enzyme preparation showed dose-dependent cytotoxicity against MCF-7 and HepG2 cell lines. Computational analyses supported the experimental findings, confirming stable substrate binding and structural stability of L-asparaginase. These results highlight the potential of fungal L-asparaginase as a promising candidate for biomedical applications, including antimicrobial, antibiofilm, and anticancer therapeutics.

## Data Availability

All data underlying the results are part of the manuscript and no additional source data are required.

## Abbreviations

ALL: Acute lymphoblastic leukemia
AUMC: Assiut University Mycological Centre
CLSI: Clinical and Laboratory Standards Institute
DEAE-cellulose: Diethylaminoethyl cellulose
DMEM: Dulbecco’s modified eagle medium
DMSO: Dimethyl sulfoxide
GAFF: General amber force field
L-Asn: L-asparagine
LINCS: LINear constraint solver
MD: Molecular dynamics
MHB: Muller-Hinton broth
MIC: Minimum inhibitory concentration
OFAT: One factor at a time
PBS: Phosphate-buffered saline
PDA: Potato dextrose agar
PDB: Protein data bank
PME: Particle Mesh Ewald
RMSD: Root mean square deviation
RMSF: Root mean square fluctuation
SASA: Solvent-accessible surface area
SD: Standard deviation
SDS-PAGE: Sodium dodecyl sulfate-polyacrylamide gel electrophoresis
